# Integrating cardiovascular risk biomarkers in the context of inflammaging

**DOI:** 10.18632/aging.206136

**Published:** 2024-10-14

**Authors:** Jacopo Sabbatinelli, Matilde Sbriscia, Fabiola Olivieri, Angelica Giuliani

**Affiliations:** 1 Department of Clinical and Molecular Sciences, Università Politecnica delle Marche, Ancona, Italy; 2 Clinic of Laboratory and Precision Medicine, IRCCS INRCA, Ancona, Italy; 3 Advanced Technology Center for Aging Research, IRCCS INRCA, Ancona, Italy

**Keywords:** cardiovascular disease, inflammaging, cardiac biomarkers, residual inflammatory risk

Cardiovascular diseases (CVD) are the leading cause of death globally, accounting for one-third of all mortalities. Traditionally, cardiovascular risk assessment has relied on well-established risk factors linked to atherosclerotic CVD. These include demographic factors, lifestyle habits like smoking and physical activity, and medical conditions such as diabetes, hypertension, and obesity, along with circulating markers such as non-HDL cholesterol. These risk factors have been incorporated into risk estimation tools like the SCORE2 algorithm of the European Society of Cardiology [1]. However, even after managing known modifiable risk factors, residual cardiovascular risk remains, suggesting additional unaddressed contributors. Over the years, residual cardiovascular risk has been dissected into various components – inflammatory, metabolic, prothrombotic, and proatherogenic. From a precision medicine perspective, this decomposition has facilitated the identification of clinical variables and biomarkers, enabling more effective preventive measures and treatments tailored to each individual.

The prevalence of residual inflammatory risk (RIR), typically defined by high sensitivity C-reactive protein (hs-CRP) levels ≥2 mg/L has been associated with major cardiovascular events such as myocardial infarction, stroke, and heart failure. Clinical trials, including PROVE IT-TIMI 22, IMPROVE-IT, and SPIRE-1/2, have demonstrated that about one-third of patients exhibit elevated residual inflammatory risk, making it a significant issue [2]. Currently, therapeutic tools specifically targeting residual inflammatory risk have been explored primarily in clinical trials. Trials such as CANTOS, COLCOT, LoDoCo, and LoDoCo 2 have shown promising results, with interventions like the IL-1β antagonist canakinumab and Colchicine significantly reducing recurrent cardiovascular events in individuals with residual inflammatory risk or chronic coronary disease [3].

Aging-related low-grade chronic inflammation, termed “inflammaging”, plays a crucial role in residual inflammatory risk [4]. Studies have linked residual inflammatory risk to factors like sex, obesity, blood glucose levels, and comorbidities. These risk factors converge on shared pathways, leading to chronic, low-grade inflammation, which contributes to CVD.

Several biomarkers, already in use for specific CVDs, like heart failure, or in other diseases with a significant impact on the cardiovascular system, such as type 2 diabetes and chronic kidney disease, can provide valuable information for risk assessment even in individuals without evident disease [5]. These biomarkers include markers of cardiac damage, such as high-sensitivity cardiac troponin (hs-cTn) and natriuretic peptides, which fit into this context with a dual role; they can represent both surrogate endpoints capable of early identification of subclinical cardiovascular damage and predictors of future cardiovascular events in primary and secondary prevention. Technological advances in immunoassay sensitivity have improved the ability to detect low concentrations of these markers in apparently healthy populations.

In clinical practice, high-sensitivity cardiac troponin (hs-cTn) meets the key criteria for a useful biomarker—it can be measured accurately, provides additional information beyond other biomarkers, and guides clinical decision-making. For instance, in general population screening, hs-cTn levels have been shown to decrease with interventions like statin use, exercise, and weight loss [6]. Furthermore, its relatively low cost makes hs-cTn an efficient tool for improving quality-adjusted life years by reducing invasive procedures and hospitalizations related to cardiovascular disease.

Given its strong evidence base, incorporating hs-cTn into existing cardiovascular risk estimation algorithms is an important step forward. To date, only one manufacturer has established sex-specific hs-cTnI cut-offs for risk stratification, based on data from large cohort studies [7]. These cut-offs define three risk categories: low (females <4 ng/L, males <6 ng/L), moderate (females 4–10 ng/L, males 6–12 ng/L), and high (females >10 ng/L, males >12 ng/L). According to this approach, low-risk individuals can remain under standard primary prevention. Those at moderate risk may require more intensive lifestyle changes, while high-risk individuals could benefit from both pharmacological treatments, such as statins, and specialized diagnostic tests in cardiology.

This reasoning extends beyond hs-cTn to other biomarkers, particularly in heart failure. For instance, natriuretic peptides like BNP and NT-proBNP, even at levels below the decision limits for heart failure, are associated with future heart failure and cardiovascular or all-cause mortality [8]. Longitudinal increases in NT-proBNP over time also correlate with worsening heart failure and higher mortality risk.

As automated immunoassays become more widely available, a multi-marker strategy combining hs-cTn, natriuretic peptides, and other markers becomes more feasible. This approach should not only focus on cardiac-specific markers but also consider extracardiac markers capable of describing the contribution of systemic inflammation (and inflammaging) to the deterioration of cardiac function. Among these, soluble ST2 (sST2) is recognized as an important prognostic indicator for heart failure. Specifically, high serum levels (≥35 ng/mL) of sST2 are associated with a greater risk of hospitalization and cardiovascular and all-cause mortality in heart failure and conditions leading to cardiac damage, such as type 2 diabetes [9]. It is important to note that sST2 is primarily extracardiac in origin, and its levels reflect the activation of numerous cell types, including endothelial and immune cells, in response to stress, cellular damage, and inflammation. These phenomena are present not only in heart failure but also in other age-related diseases. From this perspective, sST2 represents an ideal candidate for describing the contribution of inflammaging to the worsening of cardiac function and, ultimately, the onset of major cardiovascular events in at-risk subjects.

In addition to hs-cTn, natriuretic peptides, and sST2, numerous other biomarkers have been evaluated in heart failure and other CVD, although the evidence supporting their use is more limited and, in some cases, conflicting [10].

One key factor limiting the widespread adoption of cardiac biomarkers in primary prevention is the uncertainty around how to manage elevated results. Too often, elevated levels of cardiac biomarkers in older individuals are regarded as false positives, attributed to the aging process itself rather than being recognized as signs of actual cardiac damage. Although there is strong evidence supporting their predictive value for future CV events, their use remains largely confined to managing acute conditions, such as angina or heart failure, rather than being incorporated into routine preventive care. In this context, hs-cTn and natriuretic peptides should be viewed as equivalent to other markers of organ function or dysfunction, much like creatinine (and estimated glomerular filtration rate, eGFR), which, in contrast, are the most commonly analyzed biomarkers to monitor kidney function in the general population.

Recently, the deep multi-omic phenotyping of aging, namely ageotyping, revealed that different organs age at varying rates [11]. Therefore, it is essential to also assess biomarkers that reflect declining cardiac function and heart damage. Fortunately, such biomarkers already exist and have the necessary analytical accuracy for this purpose. The “race” for the ideal biomarker, therefore, cannot disregard a deeper understanding of those biomarkers long known to laboratorians and clinicians.

**Figure 1:**
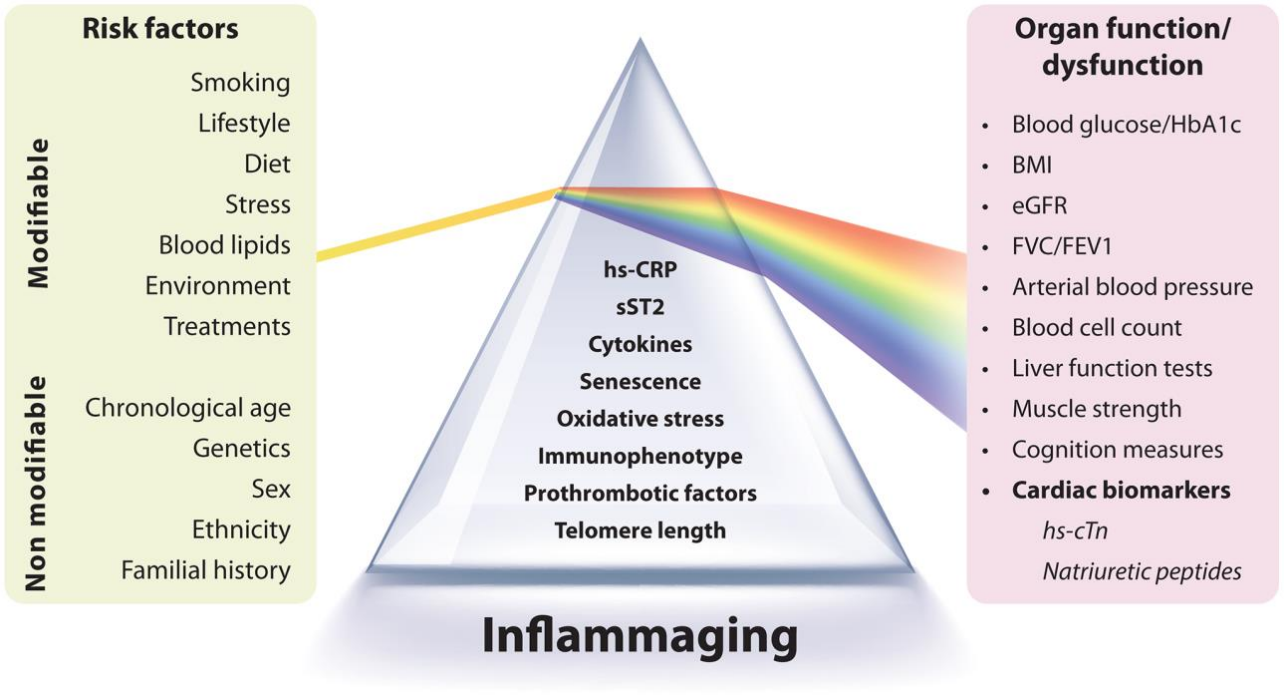
**Integration of inflammaging biomarkers with organ function markers.** This figure illustrates the interplay between biomarkers of inflammaging and markers of organ function. Inflammaging is characterized by chronic, low-grade inflammation and can be assessed using markers such as high-sensitivity C-reactive protein (hs-CRP), cytokines, cellular senescence indicators, oxidative stress, and immune profiles. Both modifiable (e.g., lifestyle, diet, smoking) and non-modifiable (e.g., genetics, sex, age) factors influence inflammaging, which contributes to age-related organ decline. Organ function is evaluated using biomarkers such as blood glucose, BMI, kidney function (eGFR), lung function (FVC/FEV1), and arterial blood pressure. The inclusion of cardiac biomarkers such as high-sensitivity cardiac troponin (hs-cTn) and natriuretic peptides (BNP, NT-proBNP) is crucial for assessing cardiovascular risk and dysfunction. These markers act as early indicators of subclinical cardiac damage and predictors of future cardiovascular events. Incorporating these cardiac biomarkers into preventive care enhances risk stratification and informs therapeutic strategies to address residual inflammatory risk, a key factor in cardiovascular disease.

Here, we advocate for a more comprehensive biomarker-based approach to CV risk that incorporates two distinct dimensions. The first group of biomarkers—such as cytokines, immune cell phenotypes, and mediators released by senescent or dysfunctional cells, which form the SASP—could provide an indication of the overall burden of inflammaging and, more broadly, residual inflammatory risk. The second group, including hs-cTn and natriuretic peptides (NPs), serves as surrogate markers that capture the impact of inflammaging on organ function (Figure 1). Crucially, biomarkers in this second group can also serve as endpoints, as numerous studies have demonstrated their responsiveness to therapeutic interventions. Establishing a mediating relationship between these two sets of biomarkers would improve our understanding of the mechanistic link between inflammaging and age-related organ dysfunction and disease.
